# The Healthy Effects of MeToo Schools: A Qualitative Analysis of Six Schools Implementing the Zero Violence Brave Club

**DOI:** 10.3390/healthcare13070739

**Published:** 2025-03-26

**Authors:** Aitor Galar, Paula Cañaveras, Cristina Pulido, Ane López de Aguileta, Garazi López de Aguileta, Ramon Flecha

**Affiliations:** 1 Department of Pedagogy, Rovira i Virgili University, 43007 Tarragona, Spain; aitor.galar@estudiants.urv.cat; 2 Department of Sociology, University of Barcelona, 08034 Barcelona, Spainglopezdeaguileta@ub.edu (G.L.d.A.); ramon.flecha@ub.edu (R.F.); 3 Department of Journalism and Communication Sciences, Autonomous University of Barcelona, 08193 Barcelona, Spain; cristina.pulido@uab.cat

**Keywords:** MeToo schools, zero violence brave club, prevention, child protection, health, children’s well-being, ECIs, evidence-based interventions, social impact

## Abstract

**Background/Objectives:** Child protection from any form of physical or mental abuse or mistreatment is a fundamental right. The scientific literature supports that evidence-based interventions, such as the “Zero Violence Brave Club”, contribute to reducing school bullying by promoting a culture of zero tolerance for violence in diverse educational contexts, regardless of socioeconomic or cultural characteristics. This research aims to analyze how the prevention model, supported by evidence-based interventions with social impacts, is implemented in six schools and to evaluate its impact on child protection and its positive effects on children’s health. This study focuses on schools that adopt a clear stance against violence and implement prevention measures aligned with evidence-based guidelines, such as those established by the recent MeToo Schools movement. **Methods:** To achieve this, fieldwork was conducted, including in-depth interviews with 13 educational community members. **Results:** The findings show that the studied schools applying SESI-based interventions prevent violence, strengthen child protection, and enhance the health and well-being of children. **Conclusions:** This research highlights the importance of implementing evidence-based programs such as the Zero Violence Brave Club (ZVBC), contributing not only to the prevention of violence but also to the improvement of students’ health and well-being, offering schools a tool to position themselves as a safe space for children.

## 1. Introduction

More than one billion children, which is half of the world’s child population, are exposed to violence every year in a variety of settings, including the digital world [1]. This exposure can lead to toxic stress, anxiety, depression, and other long-term emotional and psychological problems, significantly impacting mental and physical health throughout life [2,3,4,5]. Childhood violence is considered one of the leading preventable causes of mental illness, contributing to disorders such as post-traumatic stress disorder, borderline personality disorder, substance use disorders, sleep and eating disorders, and even suicide, which is the third leading cause of death among adolescents aged 15–19 years [1,6]. In addition to these mental health consequences, childhood violence also has serious physical health effects, including bodily injuries, sleep disturbances, gastrointestinal problems, and other health issues that negatively impact overall well-being [7].

These effects on both mental and physical health are enduring and can persist long into adulthood. A systematic review and meta-analysis of 37 studies published in The Lancet concluded that adverse childhood experiences (ACEs), including violence, have lasting effects on health in adulthood [8]. Research also shows that experiences of physical, sexual, or emotional abuse, as well as neglect, alter brain development, affecting areas such as the anterior cingulate cortex, prefrontal cortex, amygdala, and hippocampus—regions involved in emotion regulation, decision-making, and memory. These neurological changes are related to approximately 45% of psychiatric disorders that develop in childhood [9].

In the educational context, these situations hinder children’s full development, violate their fundamental rights, and compromise their quality of life. Childhood violence significantly impacts education, with victims facing a 13% probability of not graduating and higher school absence rates, especially among bullied boys and girls who have experienced sexual violence [10,11].

### 1.1. Violence in Schools and the Role of Preventing and Mitigating It

Data underscore the critical nature of this issue, establishing it as a significant public health concern. The widespread prevalence of childhood violence and its profound impacts on both physical and mental health contribute to long-term societal burdens, including increased healthcare costs and diminished economic productivity [12,13]. This highlights the necessity of preventing and eradicating child violence, as well as the need for initiatives to protect them in all vulnerable environments where they may be exposed to violence. In this study, when referring to children exposed to violence, we include those who experience domestic violence at home, those who witness violence between peers in the classroom, and those who directly suffer violence themselves.

Violence prevention in schools is not only a priority, but the school context is also an ideal environment for violence prevention. As key environments for children’s development, schools are uniquely positioned to create spaces where supportive friendships can flourish, playing a vital role in maintaining safe environments and fostering high-quality relationships among peers [14,15]. Successful prevention initiatives can contribute not only to ensuring the immediate safety of students but also to fostering environments that support healthy psychological and social development and impact the entire community. Positive social connections and powerful peer relationships have been shown to promote resilience in victims of violence across various contexts, serving as a crucial factor in moderating the long-term mental health consequences of early violence [16]. These connections help to reduce the likelihood of adolescent depression and suicide attempts, acting as a protective factor against trauma, facilitating recovery from its psychological aftermath, and enhancing the overall quality of life of these students [6,17,18].

Exposure to violence, whether direct or indirect, has been linked to lower academic performance, increased dropout rates, and long-term emotional distress, which can hinder students’ ability to reach their full potential [19]. Implementing programs that have demonstrated effectiveness in preventing violence in these institutions can significantly reduce the risk of aggression, bullying, and other forms of violence, thereby creating safer and more conducive learning environments.

The report “Achieving student well-being for all: educational contexts free of violence” [16] analyzed the prevalence and impacts of violence against children (VAC) and its relationship with educational opportunities and students’ academic performance, examining and identifying programs, supported by scientific evidence and highlighted for their social impact, that have been proven to mitigate the consequences of violence and promote student well-being.

The Zero Violence Brave Club [15,16,20] is one of the Successful Educational Actions (SEAs) highlighted. It has demonstrated its effectiveness in reducing school bullying by promoting a culture of zero tolerance for violence in diverse educational contexts, regardless of socioeconomic or cultural characteristics.

### 1.2. The MeToo Schools Movement

Some schools have adopted a selective approach in which they only allow the implementation of the best initiatives that have solid support to improve the situation. These institutions have integrated successful actions such as the Zero Violence Brave Club. In addition, the MeToo Schools movement is an example of how schools can take a comprehensive approach to combating violence and creating safer environments for students. The MeToo Schools movement is dedicated to eradicating sexual harassment in the 0–18 years age group in educational settings [21]. Officially introduced in November 2024, it was motivated by a media interview where experiences of school sexual harassment were described on one of the most popular radio stations in Catalonia, Spain (Ràdio 4) [22]. After this, the movement was created [23], leading a collective effort by adult women who have experienced sexual harassment in school environments as minors. Its mission is to ensure that no girl endures such harassment in any educational setting. To achieve this, the movement focuses, among other aspects, on identifying and highlighting actions that have helped individuals to escape harassment. In addition, one of its key aspects is supporting and disseminating research that provides scientific evidence on the social impact of harassment, particularly effective strategies for prevention and intervention.

While recognizing the efforts of various individuals, organizations, and groups working against all forms of harassment involving minors, MeToo Schools concentrates on a specific area that is often overlooked. The movement focuses on adult survivors of sexual harassment that occurred during their time as minors in school settings, which encompass both physical spaces and online environments within the educational community.

These actions have a strong impact on preventing violence, strengthening child protection, and improving children’s overall well-being and health. By adopting these measures, schools become safer spaces and communities that foster children’s integrated and positive development, preparing them for a healthier, violence-free future.

### 1.3. The Zero Violence Brave Club

The Zero Violence Brave Club (ZVBC) [24] is a successful action that empowers students to actively oppose violence by speaking up about incidents while promoting friendship and solidarity. Within the club, students develop skills to protect and stand by victims while discouraging violent behavior by disengaging from those who exhibit it. It fosters a culture of dialogic leadership, where students can openly discuss their experiences, emotions, and moral values, all within a strong commitment to creating a violence-free environment [20].

It is based on the Theory of Preventive Socialization of Gender Violence [25,26,27] and is integrated into the Dialogic Model of Conflict Prevention and Resolution [15,28,29], one of the Successful Educational Actions [30,31,32] that has proven effective in preventing violence in various contexts. The best results have been observed when it is implemented alongside other Successful Educational Actions.

One of the key aspects of the Zero Violence Brave Club is its use of the language of ethics and the language of desire [33]. By confronting aggressors or potential aggressors, they display courage that reshapes the community’s perception of desire. Aggressors appear foolish, while admiration is directed toward those who act with integrity. This interplay between ethics and desire encourages aggressors to abandon their violent behavior, leading them toward peaceful conduct and ultimately eliminating violence in this context.

The Zero Violence Brave Club should not be understood as an activity with a specific start and end time. Once implemented, the Club de Valientes continuously permeates all school activities and interactions. Although assemblies to discuss conflicts and solutions are held with varying frequencies depending on each school, the Zero Violence Brave Club is a cross-cutting activity that is an integral part of the school’s daily routine. Its aim is for students to identify situations of violence, act accordingly by reporting them, and protect the victims. Teachers and students work together to highlight the value of those who do not use violence and protect others while, at the same time, working to minimize the attraction to violent attitudes within the peer group, promoting the desirability of treating others well and rejecting violence.

This research focuses on the analysis of the specific action of the Zero Violence Brave Club as a successful action that has demonstrated a social impact in the prevention and reduction of violence in the educational environment, according to the report “Achieving student well-being for all: educational contexts free of violence” [16]. Unlike other programs, the ZVBC is backed by scientific evidence of its social impact and has, therefore, been able to demonstrate its effectiveness in promoting safer educational environments. Thus, this study aimed to collect participants’ perceptions of children’s health in six schools implementing the ZVBC model—an evidence-based prevention intervention—and to analyze its impact on child protection and its effects on children’s health, in alignment with the anti-violence guidelines of the MeToo Schools movement.

## 2. Materials and Methods

The methodology followed the communicative methodology (CM) [34] approach, which has been recommended by the European Commission for its social impact and is particularly suited for research involving vulnerable groups [25,35,36,37]. This approach transcends the traditional researcher–participant dynamic, fostering an egalitarian dialog where the researcher contributes scientific knowledge and the participant shares their lived experiences, thereby collaboratively advancing scientific understanding. The following were the hypotheses that this study aimed to explore.

**H1.**  *The implementation of the “Zero Violence Brave Club” in schools leads to a reduction in health problems related to exposure to violence.*

**H2.**  *The implementation of evidence-based interventions such as the “Zero Violence Brave Club” can strengthen child protection policies and promote a culture of zero tolerance for violence, thereby enhancing the overall well-being of students within the educational environment.*

**H3.**  *Schools that adopt evidence-based intervention programs aligned with guidelines, such as those from the MeToo Schools movement, demonstrate higher levels of child protection.*

### 2.1. Participant Selection and Data Collection

This study was conducted within the context of Spain’s education system, which operates under a decentralized structure, with regional governments having significant autonomy in determining educational policies. Spain’s general education system includes Early Childhood Education (ages 3–6), with some schools offering programs starting at age 2; Primary Education (ages 6–12); Secondary Education (ages 12–16); and Post-Compulsory Education (ages 16+), encompassing both academic and vocational tracks. Five of the six participating schools included Preschool (ages 3–6) and Primary Education (ages 6–12), although all participating teachers were involved in the Primary Education level (ages 6–12). The other school was a special education institution that also served students over the age of 18.

Although the Zero Violence Brave Club (ZVBC) is implemented not only in Early Childhood and Primary Education but also in Secondary Education, this study did not include participants from secondary school settings, except for the special education school, which served students aged 3 to 21 years old. Additionally, students in this special education school who were enrolled in the adapted basic qualification program in agriculture could attend until the age of 24.

Participants were selected through key contacts from each of the schools. These schools were chosen based on their proven and rigorous Zero Violence Brave Club implementation. Out of the 13 schools initially approached, contact was made with a representative from each institution, including teachers, principals or researchers with ties to the school. Six of the schools agreed to participate and provided one or more members of their community whom they deemed most suitable to offer valuable insights. The remaining seven schools did not respond, but no school declined the invitation to participate. The six final schools had implementation periods for the Zero Violence Brave Club ranging from 2 to 11 years. Participants varied in their familiarity with this Successful Educational Action; some had been involved since its initial implementation, while others had joined the school later and had only two years of experience in learning about and applying the program (see Table 1 for participant profiles).

The participating schools were located in the regions of the Valencian Community, Catalonia, and the Basque Country, covering the eastern and northeastern areas of Spain.

Among the 13 participants, 12 were teaching staff, including the principals of four of the six participating schools. The other participant was a mother. Although this study initially aimed to include a broader range of individuals, such as more families and students or former students over 18, the study’s management chose not to push the schools for additional participation due to the topic’s sensitivity. As a result, the participants were selected based on a voluntary initiative from the school principals.

In total, in-depth interviews were conducted with 13 participants, focusing on two key questions: (1) whether they perceived that students exposed to violence experienced health problems (and which ones if they could specify) and (2) whether they observed a reduction in these issues following the implementation of the Zero Violence Brave Club. The interviews were conducted in February 2025. The interviews were recorded using audio recordings for subsequent transcription. The interviews were conducted virtually via Zoom.

### 2.2. Data Analysis

For data analysis, all interviews were transcribed, and, in the first phase, the symptoms of physical and mental discomfort reported by the participants were identified and grouped. In the second phase, through a communicative analysis, the research team classified these elements into two categories—transformative or exclusionary—based on whether these participants’ perceptions aligned with a positive or negative impact on students’ well-being. This classification took into account the relationship between the implementation of the Brave Club, the success of the zero violence action, and the reduction or persistence of distress symptoms potentially linked to exposure to violence.

To ensure a comprehensive and consistent understanding of the data, the researchers conducted several rounds of review. Through a collaborative dialog, they refined the classification and identified two main analytic categories—psychological well-being and improved physical health structures—in the different sections of the results.

All participants provided informed consent prior to their involvement in the study, in which they were informed about the research objectives as well as their rights as participants, including the option to withdraw from the study at any time without the need for justification.

## 3. Results

This study identified various psychological and physical symptoms among the students in the participating educational centers. During the interviews, both teachers and families linked these symptoms to the students’ exposure to situations of violence. While we do not have concrete evidence to associate these health problems with exposure to violence directly, it is the participants who establish this correlation based on their experiences and observations. This study aimed to gather participants’ perceptions of the children’s health in the schools that developed a ZVBC. However, in one specific case, a participating teacher explicitly stated that a physician had attributed a student’s health problems to exposure to violence at their previous school. More importantly, we can observe a clear link between a reduction or improvement in these symptoms (regardless of their initial cause) and the implementation of the Zero Violence Brave Club in participants’ testimonies. This SEA creates a safe space where students can report violence and receive support and solidarity from their peers.

Table 2 outlines the physical and mental health symptoms identified in students exposed to violence, as reported by the study participants. Low self-esteem, regression of milestones, and sleep disorders are the most frequently reported psychological symptoms, followed by stress or anxiety and depressive mood or apathy. In terms of physical health, abdominal pain is the most reported symptom, followed equally by headaches, episodes of intense anger or impulsive outbursts, tachycardia, blackouts, and self-harm.

Participants frequently described how the distress caused by violence was not limited to the individuals directly involved but also extended to bystanders, reflecting a broader psychosocial impact. Several students who had witnessed violence—for example, in the school cafeteria—complained of headaches. It was later discovered that, when this happened, a violent incident had occurred shortly before.

In another example, two teachers explained two separate occurrences with different students. One of them began to faint after witnessing one of her closest classmates engaging in violence. Initially, they did not identify the connection, but, later, they realized that the fainting episodes occurred after witnessing incidents of violence. In the second case, the student also fainted after experiencing a distressing situation.

Monica: A clear example of students with whom we worked extensively on the Brave Club is a case where one of the students did not behave as expected (…) and how the reaction of another classmate started to, for example, if she had behavior in the dining hall, the classmate would freeze up and be even so affected that she would faint. (…) When she saw any violence again, the same thing always happened: she would faint and have tachycardia… (…) That happened to her in repeated episodes several times.

School 4, Teacher 3

Teresa: A student who fainted—the doctor said she had a non-vasovagal syncope, and the psychologist said it was related to stress (without knowing anything about what had happened the day before). So, I connected the dots: Elena was actually exposed to a violent situation she was not used to. No one has scientifically confirmed to me that this is related, but the truth is that she had never fainted before, and I do not rule out that this, for example, was a trigger that increased the likelihood of it happening.

School 4, Teacher 3

Another mother described behavioral changes in her daughter, who had special educational needs, after transferring from a mainstream school that did not implement the Zero Violence Brave Club to a special education institution where it was in place. She reported that, when conflicts arose in the latter school, the symptoms were immediately noticeable, with the child exhibiting signs such as urinary incontinence and disrupted sleep patterns.

Camilla: In Ingrid’s case, when she was attending a regular school, we noticed it more at home. Once she started at the specialized school, she gradually became calmer. But it’s also true that when she has witnessed episodes of violence (…) and has been present, we immediately noticed changes at home: she wet herself… constant flapping, repeating a specific word on her communicator… (…) In her own way, she alerts you to what has happened.

School 4, Mother 1

The teachers involved in the study identified cases of students who had been part of a school implementing the Zero Violence Brave Club and who, upon transitioning to secondary school, where conflict resolution was generally not addressed in the same way, showed a deterioration in their well-being. One teacher described how the school environment acted as a “buffer” for a student with significant difficulties, including suspected mental health issues. The student had arrived at the school with very violent behavior, coming from a home environment marked by alcoholism, parental violence, and prolonged periods of being left alone. The teacher reported a significant improvement in the child’s behavior during their time at the school but noted a regression after transitioning to a different school for secondary education. She identified that, during the time in which the child was at the school, the interventions of the Zero Violence Brave Club mitigated the symptoms that he experienced. She attributed the decline in his behavior to the lack of similar interventions at the secondary school.

Sofia: In terms of learning, there was no problem; his behavior improved significantly. However, he still occasionally displayed violent behavior and specific issues… (…) The context was a great buffer from what it could have been throughout primary school, thanks to Brave Club, Violence 0, and the fact that the school does not allow violence to take a stand and report it (…) But when he moved on to high school and, everything changed, now he is in 3rd year, (…) The behavior kept getting worse (…). At the high school, they don’t know what to do…

School 1, Teacher 1

### 3.1. Perceptions of Improved Psychological Well-Being After Zero Violence Brave Club Implementation

Implementing the Zero Violence Brave Club created a safe environment where students felt empowered to report incidents of violence, both at school and in other settings, in the studied contexts. As their confidence grew, they became more capable of disclosing personal experiences and witnessing incidents, including those at home or in extracurricular activities. Reducing violence fostered a calmer atmosphere, allowing students to thrive and alleviating distressing symptoms. Participants emphasized that the initiative boosted students’ self-esteem and sense of security, knowing that the reported situations would be addressed and that the community consistently supported and protected victims. This can be seen in the multiple examples below, which show a perception of improved psychological well-being in children.

Silvia: Last year, I had a student who it took a lot, a lot of effort to help gain confidence, and we achieved that with Brave Club, giving him the security to the point where he dared to speak on some occasions, something he hadn’t done before.

School 5, Teacher 1

Marta: When they practice it well [ZVBC], especially students who have been very beaten down because they’ve suffered violence, they come to the center as a safe space, in terms of health and well-being, and you see them doing better. They express more and verbalize more: “Here I feel safe, and I feel good,” and some even in terms of mental health. That’s something we do see.

School 4, Teacher 1

Teachers emphasized how these safe and protective spaces fostered a strong sense of friendship, significantly contributing to overall well-being. This, in turn, had a positive impact on students’ health, as evidenced by the reduction in the specific psychological health symptoms that they previously exhibited.

Marta: It’s a very powerful change when they’ve seen it here in the environment: here, violence is not tolerated, they are very clear about that, and then the relationship changes (…) What it really allows our students to do is create networks; the club, feeling part of it, and the satisfaction helps to create positive relationships, and many improvements are made in the club. In the school, increasing the spaces where we can talk helps us; they need it, too.

School 4, Teacher 1

Sofia: He was happy and doing well at school, and he had friends who protected him… but it’s true that sometimes you would see or he would express or show… “Well, on the street, I was the last one to arrive and they ran off” (…) All of that was reported, both by him and his friends, to protect him, and that’s what we know generates resilience through those friendships.

School 1, Teacher 1

This approach extends beyond the school environment, as illustrated by examples shared by two teachers. One case involved an incident during an extracurricular activity between a student and a peer from the same school. In another instance, a teacher described how the school’s zero-tolerance stance on violence encouraged a mother, who had previously avoided the school due to language barriers, to become more involved after being invited to participate in various activities. As she grew more engaged, she ultimately found the courage to report that both she and her child were experiencing domestic abuse, with her child enduring physical violence while she suffered psychological abuse.

Sofia: Two years ago, a child from Ukraine arrived due to the war, a refugee who didn’t know the language and hadn’t been schooled before. The father told us that in Ukraine, he had been undergoing tests for mental health issues, anger outbursts… and they didn’t really know what was happening to him (…) We started to realize something was wrong at home; sometimes, the father tolerated everything and other times, he was too strict (…) The child finished the school year much better; he wasn’t a “normal” child, but he was much better. (…) The mother started coming to class for interactive groups because she didn’t speak the language at all, but we insisted that she come, and we began to identify things (…) In May, she was accompanied to the center by another mother to explain that the father was mistreating the child and, well, also the mother (not physically, but still), and with the child, it was physical. Then, the mother and child left the house because they saw only one solution: to go to Ukraine. She was very grateful for that.

School 1, Teacher 1

### 3.2. Perceptions of Improved Physical Well-Being After Zero Violence Brave Club Implementation

The participants also identified improvements in health associated with physical well-being, including a reduction in negative symptoms and an increase in overall well-being for the students. The stories described children who experienced issues such as stomach aches or headaches; on one occasion, it was even noted that the doctor identified these symptoms as being caused by the violent environment that the student had been in at a previous school. All these symptoms improved as the children transferred to a new school where the Zero Violence Brave Club was implemented or as the program progressed throughout the school year.

Elisa: She started because at the beginning of the year, she had a lot of stomach pains, and her parents took her to the doctor several times. The doctors told them that there was nothing physically wrong, but she didn’t want to eat because her stomach hurt… (…) and her parents were worried because they couldn’t find anything physical. As a result of all this, the girl was finally able to express that it was because some classmates were… what she considered bullying: they ignored her, didn’t want to talk to her, treated her badly… Well, when we intervened, the girl’s health improved, and she no longer has those stomach pains… the family is more at ease now…

School 6, Teacher 2

Aaron: The headaches and stomach pains decrease each year. When Brave Club is put into action, this drops significantly (…) At the beginning of the school year, it’s very typical that it happens every day (…) As the year progresses, this decreases a lot.

School 3, Teacher 1

Sofia: A student who is in 3rd year of ESO [Secondary Education]; when he finished preschool (…), he was having health problems (…), anxiety, regression of progress: no longer controlling his bladder, self-harming (…) they went to the doctor and were told it was due to the violence at school [his former school]. They transferred him to a new school [where they do apply [ZVBC]] (…) The first day when he left school, he said, “No one fought today” (…) The parents were very happy (…) All the symptoms disappeared (…) Now, he is doing well and has had normal schooling.

School 1, Teacher 1

Another teacher recounted the case of a child who refused to go to school and would complain of stomach pain every morning, as reported by his mother. This was linked to a violent situation that he was experiencing. Once he found the courage to speak out, his peers rallied around him, creating a “protective shield”. As a result, his discomfort subsided, and he no longer complained of stomach pain or exhibited resistance to attending school.

Isabel: As a result of explaining it in Brave Club, he was able to report it, measures were put in place, the classmates listened to him and created a protective shield (…) His stomach pain stopped (…) Before starting Brave Club, there were more health problems than there are now.

School 2, Teacher 1

A special education teacher working with children experiencing severe mental health challenges reported significant improvements associated with the Zero Violence Brave Club. She shared a case in which a psychiatrist monitoring a child’s progress became highly interested in the school’s approach after observing notable health improvements in the child. In another instance, the same teacher described cases where students required reduced medication dosages, many of whom were prescribed strong medications, such as diazepam, after joining the school. In some cases, physicians even recommended discontinuing medication during school hours.

Other participating teachers highlighted the impact of applying the ZVBC on students’ resilience. They observed that it not only strengthened their ability to cope with adversity but also enhanced their resistance to illnesses, ultimately leading to a reduction in health-related absenteeism.

Silvia: With last year’s group, there were several episodes, especially linking a lot of virus episodes with others… (…) It’s like a chain reaction. You notice that children who were very weak before, in many ways, become stronger, and it all connects. Because they feel more secure, they come to school happier; they resist colds more… The atmosphere changes, from one where there was more fear and insecurity to an environment where they feel safer…

School 5, Teacher 1

Silvia: The case of a girl is very much linked to the fact that she misses school a lot and gets stomach aches when there is an episode of violence in the group. Because we do Brave Club, but there are still occasional episodes, and indirectly, she ends up missing school, if not the next day, then in two days, and it’s always because she has stomach pains, because it creates a lot of tension. (…) And with another student who vomited his lunch right after eating it he also improved, and possibly one of the factors I associate with the improvement is directly linked to Brave Club, in addition to him finding meaning in coming to school. (…) With the groups I’ve had, I’ve really noticed the change, and people who used to miss a lot before now they don’t miss as much. I believe that, in many cases, it was a somatization of the situations of violence experienced, even if the person wasn’t directly involved in the violence, but because it was in the environment and there was more tension.

School 5, Teacher 1

Aaron: I remember a meeting with a father who said that the previous year, his son would say every day that he didn’t want to go to school, and every morning his stomach and head would hurt, and that year, it disappeared. It was a family in an extremely difficult situation of poverty, and the child was being bullied by 3 or 4 classmates in class.

School 3, Teacher 1

## 4. Discussion

The findings provide strong justification for the hypotheses, as detailed below (see Table 3). H1 is fully supported, as the participants reported significant improvements in students’ physical and mental health, attributing these changes to the zero-violence school climate fostered by the implementation of the Zero Violence Brave Club in the studied contexts. The testimonials highlighted reductions in symptoms such as anxiety, sleep disorders, and stress-related ailments, demonstrating the program’s positive impact on students’ overall well-being. In many cases, symptoms related to violence exposure specifically disappeared entirely, reinforcing the link between the Zero Violence Brave Club and improved student health outcomes. Although this hypothesis is fully supported, we cannot confirm that the initial health issues were directly caused by exposure to violence due to the absence of medical reports. However, the participants consistently reported a perceived improvement in health or a reduction in negative symptoms following the implementation of the ZVBC.

H2 is fully supported, as the interview data confirm that the intervention can contribute to strengthening child protection policies and fostering a school culture of zero tolerance for violence. The participants highlighted that the program not only established clear preventive measures and effective responses to violent situations but also ensured a safer and more protective educational environment. This positive impact benefited not only children exposed to violence but the entire educational community, including bystanders, with reported improvements in overall health and well-being.

H3 is fully supported, as schools that implemented evidence-based programs aligned with child protection guidelines, such as those promoted by the MeToo Schools movement, demonstrated higher levels of child protection. The testimonies highlighted the increased sense of safety among students and a decline in reported violent incidents, underscoring the effectiveness of these interventions in fostering a healthier and more secure school climate.

This study offers unique insights by capturing the firsthand experiences of educators and staff working directly with students in schools implementing the Brave Zero Violence Club. Some of these students had exhibited symptoms and health issues potentially consistent with exposure to violence. The participants reported a perceived association between the successful implementation of the intervention and improvements in health outcomes, including a reduction in long-term negative symptoms. The participants highlighted how the implementation of the Zero Violence Brave Club fosters an environment of zero tolerance for violence, consistent with previous findings [16,20], which in turn reduces anxiety and promotes well-being within the educational community. A key distinction of this research compared to prior studies is the identification of a cascading effect triggered by the application of this action. The participants’ accounts suggest that, beyond improving the school climate, the intervention fostered a heightened sense of psychological safety, encouraging students to disclose past and present acts of violence occurring not only at school but also at home and in external social settings. Notably, the participants reported cases in which the implementation of the ZVBC facilitated the identification of previously undetected domestic violence, as well as conflicts arising during after-school activities or on the way to school. This highlights the broader social impact of the program, demonstrating its reach beyond the school environment.

The participants’ accounts also underscored that the negative impact of violence is not limited to the direct victims; bystanders, too, exhibited deteriorating health symptoms, consistent with previous research. This phenomenon has been well documented in studies on domestic and gender-based violence, which highlight the harmful effects on children who witness such incidents, but these remain less explored in school settings [38,39,40]. This is particularly relevant in cases of isolating gender violence, where bystanders who take a stand against aggression face retaliation from perpetrators [41]. The participants described instances where students exhibited physical symptoms such as fainting episodes or stomach pain after witnessing violence, reinforcing the need for interventions that protect the entire school community from the negative health consequences of exposure to violence.

A particularly relevant finding is the contrast between students’ health trajectories before, during, and after exposure to the ZVBC. The participants reported that students transferring from schools where the ZVBC was not implemented exhibited higher levels of distress and health issues, which improved after their integration into ZVBC schools. Conversely, when students moved to secondary schools or institutions without the ZVBC, some educators observed the reappearance or worsening of symptoms. These findings highlight the ZVBC’s potential long-term protective effects and the need to extend evidence-based interventions beyond primary education.

Ultimately, the ZVBC is not merely a temporary intervention but a transformational practice that reshapes the school culture, ensuring that violence is neither tolerated nor normalized. Its pervasive and continuous implementation distinguishes it from other programs that operate in isolated sessions or as short-term campaigns. Instead, the ZVBC becomes a permanent and integral part of school life, influencing how students, teachers, and the broader educational community perceive, react to, and prevent violence. This contributes to an overall sense of well-being among students, reducing their exposure to health problems. Some participants even noted that the successful action, along with the support networks and friendships that it fosters, has a direct impact on students’ resilience, which some literature has identified as a key factor in recovery across different contexts [42,43]. In some cases, students displayed an improved capacity to cope with adversity and violence, with reports suggesting increased resistance to illnesses to which they had previously been more vulnerable.

As the issue of child violence gains visibility, social media and advocacy campaigns have led to the proliferation of interventions, some of which lack scientific validation. This study reinforces the importance of only implementing programs based on scientific evidence of their social impact to ensure that resources are allocated to interventions proven to protect children and improve their well-being. Unlike programs that have not demonstrated a social impact through scientific evidence, the ZVBC has been recognized for its proven results, making it a reliable and scalable model for schools seeking to create safer educational environments.

By aligning with initiatives such as the MeToo Schools movement, which promotes scientifically validated actions, the ZVBC contributes to the broader goal of ensuring that every child learns in an environment free from violence and its harmful consequences. This study highlights the urgent need for schools and policymakers to prioritize interventions that are not only well intentioned but also rigorously tested and proven effective in protecting children’s rights and fostering their long-term well-being.

### Limitations and Future Lines of Research

This study is based on the perceptions of members of the educational community, mainly teachers. While the participants associated symptoms of poor mental and physical health with exposure to violence—whether at school, at home, or in other environments—this was based on the perceptions of the participants of the study. This study did not include medical reports or clinical diagnoses to substantiate these associations.

On the other hand, since this study did not include a comparative design, it was not possible to prove that schools implementing evidence-based interventions such as the Zero Violence Brave Club show a significant reduction in school violence and bullying compared to schools that do not implement similar programs or implement others that are not based on scientific evidence of their social impact. Without a comparative analysis, this study’s results only reflect the effects observed in schools applying the intervention, without allowing for conclusions that can be generalized or compared to schools that do not follow the program. In addition, only six schools participated in the research, which represents a limitation, as there are many schools that apply the Zero Violence Brave Club; however, these six were specifically selected because there was evidence that they applied the successful intervention rigorously and effectively.

However, a consistent finding in all cases was the reported improvement in these symptoms over time in the schools that applied the Zero Violence Brave Club, both in specific student cases and in the overall progress of the school climate. This observation suggests a possible positive impact of SA on student well-being, although more research is needed to confirm and quantify this effect.

A key future research direction would be to expand the range of participants to include more diverse voices within the educational community and to complement qualitative findings with medical records. This would strengthen the evidence base and allow for a more robust analysis of the relationship between violence exposure and health outcomes specifically related to the implementation of the Zero Violence Brave Club. Additionally, a longitudinal study tracking children who enter schools with health issues and monitoring their progress over time in Zero Violence Brave Club schools would provide valuable insights into this successful action’s long-term effectiveness.

It is important to note that this was a qualitative study and did not include a representative sample of the broader population. While it provides valuable insights into the participants’ experiences and perceptions, the findings should be interpreted with these methodological constraints in mind.

## 5. Conclusions

There are many programs aimed at violence prevention in schools, but not all of them have demonstrated a social impact in their implementation. The Zero Violence Brave Club (ZVBC) stands out not only for its contribution to preventing and eradicating violence in schools but also because this research gathered accounts from school staff who associated its implementation with improvements in various health symptoms—symptoms that may have been related to exposure to violence, such as anxiety, stress, and sleep disorders.

These findings align with the principles of the MeToo Schools movement, which prioritizes the adoption and implementation of actions grounded in evidence of a social impact. The ZVBC is an example of how schools can become safe spaces, acting as a shield against violence not only within the school environment but also in the various contexts with which children come into contact.

Non-evidence-based programs not only lack assurance of the required improvements but also jeopardize the safety and well-being of the most vulnerable children, particularly those already exposed to violence. This research highlights the importance of opting for evidence-based interventions such as the ZVBC to ensure that schools are safe and supportive environments for all students.

## Figures and Tables

**Table 1:** School profiles, implementation durations and participant demographics.

School Profile	Years Implementing ZVBC	Location	Teacher	Family
School l Students from 3 to 12 years old	10 years	Valencian C.	1	
School 2 Students from 3 to 12 years old	9 years	Valencian C.	3	
School 3 Students from 2 to 12 years old	2 years	Basque Country	1	
School 4 Students from 3 to 21 years old	9 years	Valencian C.	4	1
School 5 Students from 3 to 12 years old	3 years	Valencian C.	1	
School 6 Students from 3 to 12 years old	11 years	Catalonia	2	
Total = 13			12	

**Table 2:** Most frequently reported physical and mental health symptoms in students exposed.

Psychological Symptom	Recurrence	Physical Symptom	Recurrence
Stress/anxiety	3	Episodes of intense anger or impulsive outbursts	2
Regression in developmental milestones (e.g., loss of sphincter control, language, etc.)	4	Headaches	3
Low self-esteem	5	Abdominal pain	5
Sleep disorders	4	Vomiting	1
Depressive mood/apathy	3	Tachycardia	2
Loss of appetite	2	Blackouts	2
Hyperactivity	1	Self-harm	2
Schizophrenia	1		
Suicidal ideation	1		

**Table 3:** Consistency table: relationship between research hypotheses, methods, results and discussion.

Research Hypothesis	Methodology	Results	Discussion
H1. The implementation of the “Zero Violence Brave Club” in schools leads to a reduction in health problems related to exposure to violence.	Analysis of reported symptoms. Included changes in students transitioning between schools with and without ZVBC—both those arriving from non-ZVBC schools and those leaving ZVBC-implementing schools.	Participants reported perceived improvements in students’ mental and physical health, including reductions in anxiety, sleep disorders, and stress-related symptoms. Some symptoms associated with exposure to violence reportedly disappeared entirely.	Participants reported improvements in both physical and mental health. These positive changes or reductions in negative symptoms were observed in students who directly experienced violence (in any context), as well as in those who witnessed it.
H2. The implementation of evidence-based interventions such as the “Zero Violence Brave Club” can strengthens child protection policies and promotes a culture of zero tolerance for violence, thereby enhancing the overall well-being of students within the educational environment.	Interviews were analyzed through a communicative approach, identifying how the ZVBC influenced the school culture. Schools with different lengths of implementation (2 to 11 years) were included.	Participants perceived the ZVBC as a tool that strengthened the school’s anti-violence culture, creating a safer environment for students and the entire school community. Its effectiveness in supporting child protection policies lies in its clear stance and zero-tolerance approach to violence.	Evidence indicates that the ZVBC strengthens child protection policies and promotes a culture of zero tolerance for violence. Participants emphasized the need for scientifically validated prevention strategies, particularly given the observed pattern: children transitioning to ZVBC-implementing schools show health improvements, while those moving to non-implementing schools experience a decline in physical and mental well-being.
H3. Schools that adopt evidence-based intervention programs aligned with guidelines, such as those from the MeToo Schools movement, demonstrate higher levels of child protection.	Selection of schools with rigorous ZVBC implementation. Cross-case analysis of schools following evidence-based interventions.	Participating schools implementing the ZVBC demonstrated strong child protection measures. Participants highlighted the program’s role in encouraging children to disclose violence beyond the school setting, enabling the implementation of protective measures.	Results align with the MeToo Schools movement’s principle of prioritizing interventions with a scientifically proven social impact. This study reinforces the need to select prevention programs based on scientific evidence indicating that they have demonstrated a social impact.

## Data Availability

The raw data supporting the conclusions of this article will be made available by the authors upon request.

## Abbreviations

The following abbreviations are used in this manuscript:

SESI	Scientific Evidence of Social Impact
SEAs	Successful Educational Action(s)
SAs	Successful Action(s)
ACEs	Adverse Childhood Experiences
VAC	Violence Against Children
ZVBC	Zero Violence Brave Club
